# The Complex Myeloid Network of the Liver with Diverse Functional Capacity at Steady State and in Inflammation

**DOI:** 10.3389/fimmu.2015.00179

**Published:** 2015-04-20

**Authors:** Christoph Eckert, Niklas Klein, Miroslaw Kornek, Veronika Lukacs-Kornek

**Affiliations:** ^1^Department of Medicine II, Saarland University Medical Center, Homburg, Germany

**Keywords:** dendritic cells, Kupffer cells, liver fibrosis, NASH, inflammatory monocytes

## Abstract

In recent years, it has been an explosion of information regarding the role of various myeloid cells in liver pathology. Macrophages and dendritic cell (DC) play crucial roles in multiple chronic liver diseases such as fibrosis and non-alcoholic fatty liver disease (NAFLD). The complexity of myeloid cell populations and the missing exclusive marker combination make the interpretation of the data often extremely difficult. The current review aims to summarize the multiple roles of macrophages and DCs in chronic liver diseases, especially pointing out how these cells influence liver immune and parenchymal cells thereby altering liver function and pathology. Moreover, the review outlines the currently known marker combinations for the identification of these cell populations for the study of their role in liver immunology.

## Liver as an Immune Milieu

The liver functions as a metabolic center to ensure the proper processing of nutrients and the clearance of toxins; yet, plays multiple roles in systemic immune responses and in immune surveillance. The liver receives blood from both the systemic circulation and the intestine that mixes within the liver sinusoids (1). Approximately, two-third of the hepatic blood flow procures from the oxygen rich arteria hepatica and one-third is from the vena porta carrying microbial and food-derived antigens and molecules (1, 2). The mixed blood travels through the sinusoids that are specialized blood vessels lined by the liver sinusoidal endothelial cells (LSECs). LSECs assemble a discontinuous endothelium that is in contact with various passenger and organ-resident immune cells (3). Besides LSECs, the liver contains other parenchymal cells such as hepatocytes and hepatic stellate cells (HSCs). The activation status and extracellular matrix production of HSCs are critical for the progression of multiple liver diseases (4, 5). Importantly, these liver parenchymal cells interact with the variety of immune cells, influence memory T cells, respond to danger signals, and additionally take on the role of antigen presenting cells (APCs) within the liver (6, 7). As APCs, they present antigens in the context of immunosuppressive cytokines and inhibitory surface molecules resulting largely in tolerance (6, 7). The liver also encompasses large populations of hematopoetic cells such as innate lymphocytes (NK, NKT cells, and γδT cells) and myeloid cells [dendritic cells (DCs) and macrophages] (6). Multiple cross-talks exist between hematopoetic cells and liver parenchymal cells at steady state and during injury. This review focuses on the physiological and pathological roles of liver DCs and macrophages paying special attention to chronic liver diseases such as fibrosis and non-alcoholic fatty liver disease (NAFLD).

## Liver Dendritic Cells

Dendritic cells are present in all tissues and represent the major APCS within the body (8). They constantly sense their environment and capable of recognizing pathogens and various danger signals. Activation of DCs results in their maturation toward several functionally distinct “effector DCs” (9) that drive T cell responses, such as T helper cell differentiation, induction of CTL, and T cell tolerance (9). Additionally, DCs communicate with innate lymphocytes (e.g., NK, NKT cells), therefore, can influence both innate and adaptive immune responses (8).

Murine liver DC population, similarly as in most non-lymphoid organs (except the lamina propria and dermis), consists of three types of DCs (Table 1): the cDC1s (classical type 1 DCs), the cDC2s (classical type 2 DCs), and pDCs (10, 11). Despite of this categorization, in most liver studies, DCs are evaluated as either CD11c^+^ or MHCII^+^ cells. Although neither of the molecules pinpoint exclusively DCs, using these markers liver DCs are primarily located within the portal area and rarely scattered within the parenchyma (6). The cDC1 cells resemble lymphoid tissue CD8^+^ DCs, show migratory capacity in various non-lymphoid organs, and can efficiently cross-present cell-associated antigens (10, 11). Although the role of DC migration in liver pathology has not been explored in details, antigen injected or targeted to the liver reaches the draining LN and induces T cell activation (12, 13). Additionally, migratory DCs could be identified within the portal lymphatic vessels in electronmicroscopy analyses (1, 14).

**Table 1:** **Summary of DC and macrophage population in healthy and injured liver**.

Cell types	Murine	Human	Reference
**Dendritic cells**
Classical Type 1 DCs (cDC1)	CD45^+^ PDCA1^−^CD11c^+^ CD11b^−^CD103^+^ MHCII^+^ Langerin^+∕−^F4/80^−^CX3CR1^−^	CD45^+^ HLA-DR^+^ CD141^+^ CD123^−^CD11c^+^ CD14^−^	(8, 15)
Classical Type 2 DCs (cDC2)	CD45^+^ PDCA1^−^CD11c^+^ CD11b^+^ CD103^−^MHCII^+^ F4/80^+∕−^Langerin^−^CX3CR1^+^	CD45^+^ HLA-DR^+^ CD1c^+^ CD123^−^CD11c^+^ CD141^−^CD14^+^	(10, 15–17)
pDCs	CD45^+^ PDCA1^+^ CD11c^+^	HLA-DR^+^ CD123^+^ CD11c^−^CD303^+^ CD304^+^	(17)
pre-DCs	CD45^+^ CD11c^+^ MHCII^−^Flt3^+∕−^	ND	(18)
**Macrophages**
KCs	CD68^+^ F4/80^+^ CD11b^low^ Ly6C^low^ Ly6G^−^TLR4^+^ TLR9^+^	CD68^+^	(19–22)
Ly6C^hi^ classical monocytes	F4/80^+^ CD11b^hi^ Ly6C^hi^ Gr1^+^ CX3CR1^+^ CCR2^+^	CD14^hi^ CD16^−^	(23, 24)
M1 inflammatory macrophages/monocytes	F4/80^+^ CCR9^+^ iNOS+ galectin-3^+^	CD14^+^ CD16^+^(it is not yet clarified how they differ from non-classical monocytes)	(25–27)
Restorative macrophages	F4/80^+^ CD11b^low^ Ly6C^low^	CD14^+^ CD16^+^	(24, 26, 28)

*DCs and macrophages are classified according to the recently suggested nomenclature based on the ontogeny of these cells (15). The M1 type monocyte/macrophage population present in liver injury and the restorative macrophages during resolution could not be incorporated in this nomenclature as their origin and relation to monocytes and resident liver macrophages (KCs) need further clarification in the future. DCs, dendritic cells; KCs, Kupffer cells; pDCs, plasmacytoid DCs; pre-DCs, precursor DCs*.

The cDC2s within non-lymphoid organs are heterogeneous and partially monocyte lineage derived (10, 11). Their specific role is less understood in non-lymphoid organs, involving the liver as well. While the development of this subset depends in most non-lymphoid organs on the presence of FLT3L and M-CSF, in the liver, these cells are not exclusively dependent on these growth factors but yet on an unidentified molecule (10). The liver, similarly to other non-lymphoid tissues, contains not only fully differentiated DCs but precursor DC population as well. From these pre-DCs, either FLT3L or GM-CSF can induce liver DC development bestowing DC homeostasis *in situ* (29, 30).

Functionally, CD11c^+^ cells isolated from healthy mouse liver are less mature, have lower capacity to endocytose antigen, and induce less efficient allogenic T cell activation as secondary lymphoid organ (SLO)-derived DCs (31, 32). The inhibitory/tolerogenic capacity of liver DCs could be attributed to the specific microenvironment provided by parenchymal cells of the liver. Fibroblastic and VCAM^+^ cells derived from the liver could induce hematopoetic progenitor cells to differentiate toward tolerogenic DCs *in vitro* that can inhibit experimental autoimmune hepatitis (33). It is assumed that circulatory DCs during their translocation within the liver sinusoids toward the lymphatics receive such tolerogenic education from liver parenchymal cells (14, 34). Yet, its *in vivo* relevance needs to be elucidated.

Freshly isolated murine liver CD11c^+^ cells promote Th2 rather than Th1 T cell differentiation and via interacting with NK cells induce regulatory T cell (Treg) development (35, 36). Moreover, liver DCs produce increased amount of IL-10, IL-27 but less IL-12 upon LPS stimuli (37, 38). This hyporesponsive behavior toward TLR stimuli, known as endotoxin tolerance, involves LPS/TLR4 but also extends toward other TLRs (6). This is especially important, as the liver is constantly exposed to gut derived microbial products. The breakdown in this tolerance could be observed in colitis where pro-inflammatory DC/macrophage population expands within the liver due to the increased amount of bacterial products present in the portal blood. This creates an inflammatory environment in the liver despite the absence of direct liver damage (39). The tolerant state toward TLRs is an active process and involves the action of various negative regulators of the TLR signaling pathway (6). Interestingly, under steady state, liver DCs rather respond to ECM stimuli (collagen-type I, laminin, fibronectin) that induces MHC-II upregulation and maturation of GM-CSF expanded liver DCs *in vitro* (40).

In humans, the cDC2 cells (CD11c^+^ BDCA1^+^) are the most abundant in the liver and they exhibit similar immature, tolerogenic capacity as their murine counterpart (16, 41) (Table 1). The cDC1 cell population that expresses CD141^+^ has been recently identified as a counterpart of murine CD8α^+^ cells (42). These cells induce pro-inflammatory allogeneic MLRs, resulting in IFN-γ and IL-17 production by activated T cells (17). Importantly, as opposite to cDC2s and pDCs, cDC1s (identified in the study as CD141^+^ cells) were markedly decreased during liver diseases but among the DC-subsets produced the highest level of IFN-λ (17). It is possible that functional differences are reflected among the DC subsets and each subset represents different aspects of liver immunity and tolerance. In line with this, a classification of murine liver DCs according to their lipid content distinguishes between immunogenic and tolerogenic liver DCs. Due to their acetyl-CoA carboxylase activity, HL-DCs (high lipid DCs) mount strong immunogenic CTLs while the LL-DCs (low lipid DCs) with low lipid content are tolerogenic (43). Notably, the marker combinations used for this study showed that both HL-DCs and LL-DCs include multiple DC-subsets distinguished by currently known surface markers and were not restricted to one specific subset. Novel surface molecules are needed to specifically explore their functional diversity.

pDCs are the major source of type-I IFN, regulate NK cell activity, and play important role in the induction of antiviral immunity (44, 45). The murine liver is especially rich in pDCs; yet, the human counterpart contains a smaller proportion of this population among all DCs (17) (Table 1). Under steady state condition, pDCs express low level of costimulatory molecules, are weak T cell stimulators, and induce apoptosis in activated T cells in a Treg dependent manner (46). Later could indicate a cellular interplay between pDCs and Tregs in the liver microenvironment in order to maintain the tolerogenic milieu. Accordingly, pDCs can induce efficient CD4 and CD8 T cell tolerance to orally administered antigens (47).

Microbial products, such as muramyl dipeptide present in the portal blood, upregulate PDL-1 in pDCs and reduce their response to TLR9 stimuli (48). This is another example for the TLR-mediated hyporesponsiveness (“endotoxin tolerance”) in the liver. Strikingly, upon FLT3L treatment, the expanded liver pDCs display strong immunostimulatory properties (49). It is unclear whether this could be due to the expansion of a specific subpopulation of pDCs, or their modified interaction with Tregs, or the result of the complete rearrangement in the myeloid cell compartment, and the consequent imbalance in the tolerogenic milieu.

Taken together, the multiple DC subsets within the liver participate in guarding the tolerogenic environment and primarily skewed toward suppressing T cell responses and toward induction of Tregs. While DCs are the main APCs and inducers of T cell immunity in SLOs, within the liver environment the question still remains: how immunity can be induced by DCs in such suppressive microenvironment? Induction of immunity might be attributed to special DC subpopultaions such as the CD141^+^ cDC1s in humans (17) and the CD103^+^ cDC1s in mice (50). Moreover, the appearance of novel DC population, such as monocyte-derived DCs present in iMATEs (intrahepatic myeloid-cell aggregates for T cell population expansion), participates in efficient CLT expansion within the liver (51). Alternatively, immunity is induced by migratory DCs reaching the draining LN, thus, outside of the liver suppressive environment. In line with this, antigen specifically expressed in draining LN results in hepatitis inducing CD8 T cell activation, while the same antigen within the liver induces tolerance (52). Additionally, the liver can provide newly formed structures for T cell activation resulting in immunity. Portal tract associated lymphatic structures (PALPs) during *Propionibacterium acnes* granuloma formation and tertiary lymphoid structures in biliary cirrhosis represent locations where possible T and B cell activation takes place, respectively (53, 54).

## Kupffer Cells – Resident Macrophage Population of the Liver

Kupffer cells (KCs) are tissue resident macrophages and they represent the largest hematopoetic cell population within the liver. They arise from yolk sac during fetal development (55), adjust themselves to the local microenvironment (56, 57), and renew their population at steady state locally throughout adult life with no or minimal contribution of hematopoetic progenitors or blood monocytes (58–60). In mice, KCs can be distinguished from monocytes among the F4/80^+^ cells as Ly6C^low^ CD11b^low^ cell population (20, 21) (Table 1) and possess functional specifications according to their positioning within the sinusoid (61). Recent study could distinguish two KC functional groups: the one with higher phagocytosis capacity and the one with preference toward cytokine production (61, 62). Additionally, macrophages are functionally grouped into two classes M1 and M2. While such plain classification is questionable and often overstated, still provide a simple but distinguishable concept for functional categorization of these cells. M1 (termed classically activated) macrophages are pro-inflammatory, while the M2 (termed alternatively activated) macrophages are suppressive and involved in cellular repair (63). According to this, KCs belong to the M2 type of cells and play fundamental role in homeostasis, immune surveillance, and tissue repair (63).

Their importance as tolerogenic APCs in the liver microenvironment is demonstrated in liver transplantation where they prolong allograft survival (31). At steady state, they inhibit DC mediated T cell activation within the sinusoids and presentation of high affinity peptide by KCs results in deletional CD8 T cell tolerance (6, 64). Furthermore, they promote the suppressive capacity of Tregs toward hepatic antigens (65, 66).

As all tissue resident macrophages, KCs express a wide repertoire of receptors for the recognition of pathogens and danger signals such as Toll-like receptors, members of the inflammasome, and scavenger receptors (31). In the presence of TLR ligands such as LPS and CpG, KCs become immunogenic, and can induce CD8 T cell activation, and the generation of efficient CLT response (67, 68). Thus, during liver infection, they support the development of antimicrobial T cell responses. Unfortunately, KCs induce efficient CTL against antigens from the systemic circulation such as the case in influenza infection (69). This CTL response results in bystander hepatitis, often accompanying systemic viral infections. Besides CD8 T cell responses, recent study describes naive CD4 T cell activation in the murine liver by antigens expressed in hepatocytes. This process is independent from lymphoid tissue but dependent on clodronate-sensitive liver APC population possibly involving KCs as well (70). Thus, KCs participate in the generation of both CD4 and CD8 T cell responses.

Using their scavenger receptor repertoire, KCs are involved in the clearance of apoptotic cell debris and central to iron homeostasis (71). KCs interact with multiple immune cells within the sinusoids, such as Tregs, DCs, DC precursors, and innate lymphocytes (7, 53, 72, 73). After recognizing any danger signals, KCs primarily drive the influx of inflammatory leukocytes such as neutrophils and monocytes (63). Thus, KCs function as sentinels and central orchestrators of cellular processes in healthy and injured liver. Additionally, while they support the tolerogenic milieu within the liver, their presence also ensures the protection of the liver during pathogen invasion.

## Tools to Study Liver DCs and Macrophages

In order to characterize the specific physiological and pathological roles of DCs and macrophages, various animal models, tools have been developed (Table 2) Among these models, there are mouse lines deficient in transcription factors that are responsible for the development of one or multiple subsets of myeloid cells. Due to the multiple cell types affected in these models, the broader impact of each of these genes makes it difficult to unequivocally pinpoint subset specific functions. Nevertheless, these transgenic animals helped significantly to establish broader understanding of macrophage and DC development and their role under steady state and inflammation (63, 74). However, just few of these models have been evaluated so far in fibrosis and non-alcoholic steatohepatitis (NASH) models (Table 2) but also in liver immunology. This might extend in the future as genetic model lacking cDC1s have just recently demonstrated that cDC1s respond to hepatotropic viral infection and are keys in the induction of anti-viral CD8 T cell response *in situ* (50).

**Table 2:** **Summary of the available models to study liver macrophages and DCs**.

Animal model	Cell types affected	Liver fibrosis/NASH studies
**Transcription factors**
Cfsr1^op/op^, Cfsr1^−/−^, Csfr2^−/−^	Macrophages, monocytes, some DCs, granulocytes	ND
Batf3^−/−^, ID2^−/−^, IRF8^−/−^	CD8^+^ DCs, CD103^+^ DC	ND
Flt3L^−/−^, injection of FLT3L	CD8^+^ CD11b^−^, CD11b^+^ DCs, pDCs	(75)
IRF2^−/−^, IRF4^−/−^	CD8^−^CD11b^+^ DCs	ND
**DTR system**
CD11c-DTR-short promoter-long promoter	DCs, plasmablast, some activate CD8 T cells, marginal zone macrophages, alveolar macrophages, some B cells	(75–78)
	All above + some NK and NKT, pDCs, monocyte-derived DCs	
CD11b-DTR	Neutrophils, monocytes, eosinophils, macrophages, some DCs	(28, 79, 80)
CD169-DTR	Splenic MM macrophages, LN macrophages, BM macrophages, KC	ND
Langerin-DTR	Langerin^+^ dermal DCs, langerhans cells, some CD8^+^DCs, and some CD103^+^ DCs	ND
Zbtb46-DTR	DCs and DC committed progenitors, monocytes (IL-4 and GM-CSF)	ND
**Clodronate liposome mediated cell depletion**	Macrophages, some DCs, monocytes	(81–86)
**Reporter/Cre mouse lines**
CX3CR1-GFP	Macrophages, monocytes, some DCs	(87, 88)
Cfsr1-GFP (MacGreen)	Macrophages, monocytes, some DCs	
Lyz2-GFP/Lyz2-Cre	Macrophages, granulocytes	
Cfsr1-GFP	Macrophages, monocytes, some DCs	
CCR2-RFP	Monocytes, macrophages, memory T cells	
MHCII-EGFP	Macrophages, DC, B cells	
CD11c-YFP/CD11c Cre	See above	
Langerin-GFP	See above	
DNGR-1-GFP	DCs, pre-DCs	

*ND, non-determined; DCs, dendritic cells; MM, marginal zone*.

The most frequently used cell-depleting tools in liver immunology are the clodronate liposome mediated depletion of mononuclear cells and the CD11c-/CD11b-DTR (diphtheria toxin receptor) transgenic system (87, 89). Clodronate-encapsulated liposomes are taken up by mononuclear cells and the clodronate bisphosphonate within the cell induces apoptosis that results in depletion of the phagocytic cell population. Multiple phagocytic cell types are affected using this depletion method such as KCs, macrophages, and some members of the DC population as well. Since more than one cell types are affected, the effects can be extrapolated to a group of cells and not to individual subtypes (89). Additionally, the release of inflammatory mediators (such as TNF) has been associated with this type of cell depletion further complicating the interpretation of experimental results (90).

The other widely used tool for liver biology is the CD11c-DTR-based depletion system. Here, the human diphtheria toxin receptor is expressed under the CD11c promoter and administration of diphtheria toxin results in the depletion of CD11c^+^ cells. This model is used to dissect the role of conventional DCs. The major disadvantage in this system is that multiple cell types are affected such as marginal zone macrophages, monocytes, activated CD8 T cells, NK cells, and plasmacytoid DCs (89). Two different CD11c-DTR mouse lines have been generated: the one encompassing only a short piece of the CD11c promoter (3) and the one with the full-length promoter (91). Although, they differ in the list of affected cell types, they gave important insights in the role of CD11c^+^ cells in liver immunology (Table 2). Novel DTR tools have been developed in recent years that aim to restrict the expression of DTR more specifically to DCs. The zbtb-46-DTR model uses the transcription factor zbtb46 that is exclusively expressed by DCs and DC-committed precursors (92). Unfortunately, zbtb46 is upregulated in monocytes stimulated with GM-CSF and IL-4, suggesting some limitations to this promoter (93, 94). Another promising promoter is the DC NK lectin group receptor-1 (DNGR-1) that seems to be highly restricted to the DC linage (95). Not only for DCs but also for the study of macrophages, the perfect targeting tool still needs to be developed. The primary tool for analyzing macrophages is the CD11b-DTR system. However, CD11b is a widely expressed marker among multiple immune cell types causing caveat for the interpretation of cell types using this model for understanding liver diseases (Table 2) (87).

To follow myeloid cells *in situ* using *in vivo* imaging, flow cytometry or microscopy multiple reporter mouse models have been developed. The promoters from different molecules such as e.g., CD11c, Csfr1, CCR2, MHC-II, or CX3CR1 were used to generate these animal models (88) (Table 2). These models have their own limitations according to their expression profile that have been reviewed elsewhere (88) (Table 2). Some of the promoters are also utilized to express Cre recombinase (Table 2). Crossing these Cre expressing lines with animals carrying a flox-ed gene allows the analyses of the cell specific depletion of the gene of interest. Certainly, the specificity and limitation of the models are determined by the expression pattern of the promoter used for Cre expression (87). Despite the availability of these models, only limited have been exploited for understanding specifically liver fibrosis and NASH (Table 2).

Taken together, multiple models are available to answer liver immunological questions. While each of the available models has its own limitation, the combination of these models with each other can still pinpoint important contribution of DCs and macrophages in liver pathology.

## The Role of Dendritic Cells and Macrophages in Chronic Liver Diseases

### Liver fibrosis progression

Liver fibrosis is a common endpoint of many chronic liver diseases such as viral hepatitis, primary biliary cirrhosis, alcoholic and NASH, or autoimmune liver disorder (96, 97). To investigate liver fibrogenesis, several rodent models have been developed inducing toxic (CCL_4_), biliary (bile duct ligation), oxidative (TAA-induced), or metabolic (MCD/methionine choline deficient diet induced) liver damage (96, 97). The MCD diet contains high sucrose and fat (usually 40% sucrose and 10% fat) but lacks the amino acid methionine and the small molecule choline that are essential for hepatic β-oxidation and production/secretion of very low density lipoprotein (VLDL). As a result, lipids are deposited in the liver and steatosis, and NASH develops in these animals (98).

Remarkably, even though the molecular mechanisms leading to hepatic cell death are very different, the process of fibrogenesis and the cellular components involved share common hallmarks. Such common components, that have established their role in liver fibrosis, are the macrophages and the recruited inflammatory monocytes.

Major evidence for the involvement of macrophages in liver fibrosis is demonstrated in *in vivo* depletion studies using the CD11b-DTR system and the clodronate-liposome mediated cell depletion. In CCL_4_ induced liver injury, the progression of fibrosis was attenuated in the absence of CD11b^+^ cells and the number of HSC-derived myofibroblasts was greatly reduced (79). The administration of clodronate liposomes similarly suggested that macrophages are pro-fibrogenic and affect the survival of HSCs via TNF and IL-1 induced NF-kb signaling (84, 99). Liver macrophage populations change during liver injury. One of the major changes is the recruitment of inflammatory monocytes to the injured liver and their differentiation toward tissue macrophages (24, 26, 28, 100). Resident KCs in liver injury rapidly secrete pro-inflammatory cytokines such as IL-1β, TNF, CCL2, and CCL5 resulting in recruitment of multiple immune cells involving monocytes as well. The accumulation of circulating Ly6C^hi^ monocytes within the liver is greatly dependent on CCR2/CCL2 and CCL1/CCR8 axis (100). The monocyte recruiting chemokines, however, not only originate from KCs but also from TLR-activated HSCs (101). Moreover, senescent hepatocytes and NF-kb-inducing kinase (NIK) activation in hepatocytes lead to the release of numerous chemokines (86, 102). These chemokines can influence the migration or activation state of macrophages that in turn induce hepatocyte apoptosis. Accordingly, hepatocyte-specific expression of the NIK *in vivo* triggers massive liver inflammation and hepatocyte apoptosis leading to liver fibrosis (86). Thus, the macrophage–hepatocyte cross-talk seems to greatly influence cell recruitment and the activation state of macrophages, thereby affecting the progression of liver injury. The fact that in the above study KC/macrophage depletion using clodronate reversed NIK-induced damage, also strongly suggests this.

Monocyte recruitment to the injured liver can be observed early within 24 h after the induction of CCL_4_ damage (25). These early recruited cells are CCR9^+^, colocalize and interact with CCR9^+^ HSCs (27). Furthermore, these monocyte-derived macrophages are characterized as CD11b^+^F4/80^+^iNOS^+^ cells that exhibit profibrogenic properties via promoting HSC activation, Th1 cell differentiation, and TGFβ release (25, 26). In addition to this, profibrogenic Ly6C^hi^ macrophages express PDGF, IL-13 and IL-4 that directly act on HSC derived myofibroblasts and induce ECM production (25, 26). Macrophages produce various lectins among them galectin-3 is required for TGFβ mediated myofibroblast activation and matrix production that further underline their profibrogenic capacity (103).

Another chemokine that affects the infiltrating monocytes is the fractalkine receptor (CX3CR1). Fractalkine is released by hepatocytes and HSCs during liver injury. It ensures the survival of infiltrating monocytes and influences their differentiation toward tissue macrophages (25). In the absence of CX3CR1, infiltrating monocytes develop into highly inflammatory macrophages that die early via apoptosis. This perpetuates further inflammation and recruitment of Ly6C^hi^ cells. Additionally, CX3CR1 on KCs increase their IL-10 expression and reduces their TNF and TGFβ (104). Thus, fractalkine represent a negative feedback on the extension of liver inflammation through affecting KCs and the presence and destiny of Ly6C^hi^ cells at least in the murine system. It requires future research to clarify how the changes in monocyte and macrophage subsets observed in mice are reflected in humans.

Regarding the M1–M2 classification of macrophages, during the progression phase of liver fibrosis and during fibrosis resolution, both types of cells are present in the liver side by side (105). Interestingly, based on histological analyses these M1 and M2 macrophages localize near to the fibrotic septa and could indicate further undiscovered cross-talk among these cells in liver pathology. Of note, transcriptional analyses of macrophages that are present in the resolution phase display a profile that cannot be classified according to the M1/M2 nomenclature (28).

Accumulation of macrophages within the injured liver caused just partially by the recruited monocytes and their differentiation toward tissue macrophages. There is some evidence that local multiplication of resident and monocyte-derived macrophage population contribute to this process. Ki67 staining during CCL_4_ mediated liver injury demonstrated the presence of proliferating KCs and monocyte-derived macrophages (28, 62). In most recent study, Listeria infection of the liver resulted in monocyte-derived macrophage proliferation via IL-4 and IL-33 (106). Whether these cytokines are also involved in this process during other types of liver injury and in humans as well remain to be elucidated.

Multiple animal studies reported that the number of dendritic cells, pre-DCs, and pDCs increase during the progression phase of liver fibrosis (76, 107). This raised the assumption that DCs might contribute to fibrosis progression. Using the CD11c-DTR model, it has been demonstrated that CD11c^+^ cells provide a pro-inflammatory milieu by producing IL-1β and TNF during injury (76). Moreover, isolated cells contribute to HSC survival *in vitro* suggesting a clear profibrogenic capacity of these cells (37). This phenomenon, despite of the relatively broad CD11c expression among other myeloid cells as discussed above (Tables 1 and 2), was attributed to DCs.

Another study determined using the same CD11c-DTR system that DC depletion accelerates the development of fibrosis due to their influence on angiogenesis. DCs seem to be the source of the anti-angiogenic VEGF receptor 1 (also known as sFlt-1) and thus influence the bioavailability of VEGF during fibrogenesis (78). Notably, recent study has demonstrated that VEGF^+^ inflammatory monocytes/monocyte derived macrophages colocalize with newly formed vessels in injured liver and pharmacological inhibition of CCL2 mediated recruitment of inflammatory monocytes reduces fibrosis-induced angiogenesis without affecting fibrosis progression (108). Thus, recruited monocytes/macrophages seem to counterbalance the anti-angiogenic property of DCs during fibrosis progression. Whether classical DCs or pDCs truly contribute to fibrosis progression or play other role during liver injury still remain to be clarified in the future.

### Liver fibrosis regression

During liver fibrosis, the increased production of ECM is accompanied by high expression of MMPs and the presence of collagenase activity, suggesting alterations and adjustments in the fibrotic ECM. In fact, the fibrotic ECM seems to be different biochemically than ECM produced during a steady state turnover (109). In lung fibrosis, the pathological ECM activates fibroblastic cells to build further matrix indicating a positive cross-talk between fibroblast and matrix components (109). Also during liver fibrosis, heavily cross-linked, modified ECM could be identified (110); however, it remains to be elucidated whether similar regulatory loop as in the lung operates in liver fibrosis as well.

Importantly, after removal of the noxious agents causing liver damage, fibrotic scars degrade and normal liver architecture can be restored. This process is called resolution. While this functions well in various animal models, in humans this seems to be a point of no return where fibrosis and cirrhosis progresses nonetheless (97).

In resolution, the role of macrophages has been demonstrated in multiple animal studies. Depletion of CD11b^+^ cells during fibrosis progression, as above discussed, reduced scarring while during fibrosis resolution led to a failure in matrix degradation (79, 80). This strongly suggests the dominant presence of two functionally different macrophage populations. According to this, Ramachandran et al. have identified a subset of Ly6C^low^ “restorative” macrophages during resolution (28). These cells originated from Ly6C^hi^ recruited monocytes expressed MMPs including MMP9, MMP12, and phagocytosis related genes. Importantly, based on gene expression profiling, they could not be fit in the M1/M2 macrophage classification. Moreover, phagocytosis of liposomes or cellular debris by liver macrophages could recapitulate this type of restorative phenotype (28). In addition to this, recent study demonstrated that scar associated myeloid cells attract endothelial cells to the scar tissue via VEGF and that genetic ablation of VEGF in myeloid cells resulted in the increase of MMP2 and MMP13 and decrease of TIMP1 in the liver. While macrophages have not been unequivocally identified as myeloid cells in this study, the results indicate that the myeloid cells induced angiogenesis gears the balance toward fibrolysis (111). This is in line with recent findings that demonstrated that VEGF signaling plays key role during liver fibrosis resolution. Anti-VEGF antibody treatment during resolution led to impaired tissue repair. Mechanistically, VEGF regulated endothelial permeability, monocyte recruitment, and affected the CXCL9 and MMP13 expression of scar-associated macrophages. Importantly, depletion of Cfsr1^+^ cells (including macrophages, monocytes, and DCs Table 2) impaired fibrosis resolution (112).

Based on these findings, macrophages can be grouped in profibrogenic and restorative macrophage populations beyond the M1/M2 scheme, a classification that might be much more beneficial for finding new targets for fibrosis therapy. However, multiple open questions remain concerning the balance of the heterogeneous population in liver diseases and the relation to each other. One molecule could provide a better understanding to the problem, the chemokine CX3CL1. Ramachandran et al. showed a higher expression of CX3CR1 within the restorative macrophage population, then in the profibrotic subset (28). Consistent with these findings, HSC and hepatocyte-derived fractalkine led to the induction of Arginase 1 in a mixed Kupffer-cell/macrophage cell population, a marker that has been associated with the fibrolytic macrophage subset (28, 104). Thus, an intriguing possibility is the progressive class switch between macrophage populations during fibrosis progression and regression. This possibility is underlined by the fact that the overall number of profibrogenic Ly6C^hi^ macrophages strongly decreases in resolution despite the presence of their strong proliferation activity at early time points of fibrosis regression. At the same time, the number of Ly6C^lo^ macrophages increases (28). Along this line, blocking CCL2 dependent liver infiltration by Ly6C^hi^ monocytes during fibrosis regression leads to a higher relative amount of Ly6C^lo^ macrophages (113). Moreover, the Ly6C^lo^ macrophages could be shown to be postphagocytic and seem to appear in the phase of reduced hepatocyte death, further supporting the switch concept (28). It remains to be clarified in the future how the macrophage populations interact and relate to each other. Similarly as the murine restorative macrophages in humans, this population is likely represented by the CD14^+^ CD16^+^ cells (24, 26). They display phagocytic activity but as opposite to the murine cells express a variety of pro-inflammatory and pro-fibrogenic molecules as well.

Besides macrophages, DCs have also been implicated in liver fibrosis resolution. Jiao et al. have demonstrated that depletion of CD11c^+^ cells leads to delay in fibrosis resolution and delayed clearance of activated HSCs. To more precisely pinpoint DCs in this process, adoptive transfer of purified DCs or expansion of endogenous DCs using FLT3L could accelerate regression. Moreover, DCs were the source of MMP9 and therefore seem to complement restorative macrophages in this process (75).

### Non-alcoholic fatty liver disease

Non-alcoholic fatty liver disease (NAFLD) is the hepatic manifestation of metabolic syndrome that includes hypertension, hyperlipidemia, insulin resistance, and visceral adiposity, and shows a worldwide increasing tendency among chronic liver diseases (114, 115). In most cases, the liver steatosis is mild. However, up to one-fifth of the cases progresses toward NASH that is characterized by intrahepatic inflammation, increased steatosis with hepatocellular ballooning, and often accompanied by progressive fibrosis (114, 115). NASH is prone to the development of cirrhosis and liver cancer (115). While the precise cellular and molecular mechanisms of NASH are not yet fully understood, multiple studies have investigated macrophages and DCs in this disease.

Similarly as during liver fibrogenesis, in NASH, the two main components that show alterations are the response of macrophages/KCs and the recruited inflammatory monocytes. The key role of macrophages/KCs in NASH has been demonstrated in studies where these cells were specifically depleted using gadolinium chloride or clodronate liposomes (81–83, 85). In the absence of KCs, the steatohepatitis was markedly reduced. In addition to this, KCs display an M1 TNF expressing pro-inflammatory phenotype and increase triglyceride accumulation, decrease fatty acid oxidation and insulin responsiveness of hepatocytes (82, 83). KC-derived TNF production seems to be central in NASH development, as silencing liver TNF or using TNFR1/2 deficient animals attenuate liver steatosis compared with control wild-type animals (85, 116).

Multiple triggers have been identified for KC activation and for the induction of their pro-inflammatory cytokine production in NASH. TLR4 deficient animals showed reduced liver damage and KC depletion prevented the increase in TLR4 expression during MCD diet (81). Bacterial product induced KC activation is in accordance with clinical data that demonstrate bacterial translocation in NASH patients (117). Notably, TLR4 can be triggered not only by LPS but also by free fatty acids and high mobility group box-1 protein (HMGB1) that is increased in obesity and during hepatocyte injury. Not only LPS but also translocated nucleic acids have been implicated in the development of NASH via triggering TLR9 mediated KC activation and IL-1β release (118).

Lipidomics and mass spectrometry profiling revealed that KCs accumulate toxic lipids due to the dysregulation of lipid metabolism during high fat diet. Moreover, these lipid-loaded KCs produce pro-inflammatory cytokines and chemokines (119). The balance between the M1 and M2 type of KCs seem to be a key for NASH progression. Mice fed with high-fat diet displayed a predominant M2 KC polarization, the apoptosis of M1 KCs and resistance to hepatocyte steatosis. *In vitro* experiments demonstrated that M2 macrophages release IL-10 that in return increase the sensitivity of M1 macrophages to undergo apoptosis (120).

The other hallmark of NASH is the increased monocyte recruitment to the injured liver. Activated KCs upregulate their MCP-1 expression that is the major chemokine involved in the recruitment of Ly6C^hi^ cells. These Ly6C^hi^ cells are pro-inflammatory and further perturb hepatic inflammation (85). Consequently, CCR2 deficient animals show decreased steatosis (113, 121). On the other hand, CCR2 signaling, when MCD diet is replaced with control diet, counteracts tissue resolution by perpetuating inflammation (113). This is a similar phenomenon as observed in fibrosis resolution (113).

Non-alcoholic steatohepatitis is associated with increased number of hepatic DCs identified by MHCII^+^ CD11c^+^ cells (77). Depletion of these cells using CD11c-DTR mouse model exacerbates hepatic inflammation whereas during the resolution phase delays the reconstitution of normal tissue homeostasis. Importantly, these cells take up apoptotic cells, inhibit TLR expression, T cell expansion, and cytokine production by innate cells (77). This strongly suggests DCs as an important negative regulator of NASH inflammation. As opposite to this, another study has classified CD11c^+^ cells during MCD-diet based on their lipid content (43). It remains to be clarified whether the tolerogenic LL-DC (low lipid DC) population is equivalent with the same immunoregulatory DCs in NASH as described by Henning et al. Of note, based on the surface marker expression profile of HL-DCs and LL-DCs, they rather seemed to be a part of a heterogeneous population, despite that all cells expressed various level of CD11c (43).

## Summary and Conclusion

**Figure 1:**
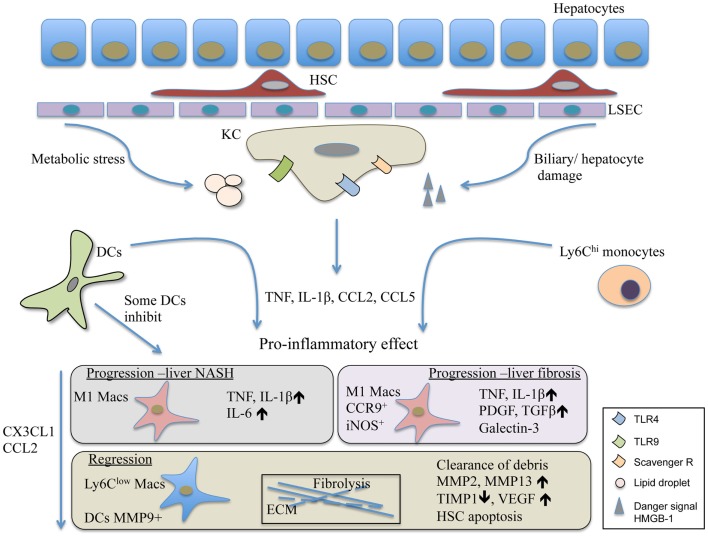
**The contribution of DCs and macrophages to the pathomechanism of liver fibrosis and NASH**. Liver injury triggers the activation of Kuppfer cells, the resident macrophage population of the liver. Their activation leads to the release of inflammatory mediators and chemokines such as TNF, IL-1β, and CCL2. This is followed by the recruitment of various immune cells involving inflammatory monocytes and DCs. The Ly6C^hi^ monocytes differentiate into M1 CCR9^+^iNOS^+^ macrophages, and together with DCs in the progression phase of liver injury, act in a pro-inflammatory manner and perpetuate inflammation. Some DCs, possibly the LL-DCs, seem to inhibit liver steatohepatitis and protect liver damage. In resolution, the Ly6C^low^ restorative macrophages together with MMP9^+^ DCs promote fibrolysis and the restoration of normal tissue architecture. HMGB-1, high mobility group box-1 protein; HSC, hepatic stellate cells; KC, Kupffer cells; LL-DC, low lipid containing DCs; LSEC, liver sinusoidal endothelial cells.

Taken together, the liver represents a unique immunological niche within the body. Its parenchymal and non-parenchymal cells guard its tolerogenic and suppressive microenvironment while supporting its sentinel task of the portal and systemic circulation (Figure 1). Most liver injuries trigger the activation of resident KC/macrophage population that rapidly releases pro-inflammatory mediators such as TNF and IL-1β. This is followed by a chain of events that seem to be commonly shared by many injuries causing NASH and leading to liver fibrosis. The response involves the alterations within the myeloid cell composition primarily affecting macrophages. Importantly, other immune cells such as DCs, neutrophils, innate cells, and activated T cell are also recruited to the injured liver and play various roles in disease progression (6, 97). The exact role of liver DCs during chronic liver injury is yet to be determined. Nevertheless, they seem to be similarly pro-inflammatory as the Ly6C^hi^ recruited monocytes. This feature is shared with infectious liver diseases; thus, it supposes to induce liver protecting immunity (2, 7). During chronic liver diseases, the overwhelming presence of pro-inflammatory immune cells together with liver damaging noxious agents eventually lead to extensive cell death and scar formation, a common outcome for chronic liver disorders. While KC activation alarms other immune cells to travel to the liver, it influences metabolic processes and survival of hepatocytes. During disease progression, Ly6C^hi^ cells seem to develop into Ly6C^lo^ restorative macrophages. These cells, if the harmful agent vanishes, lead to resolution and can restore normal tissue architecture (Figure 1). Especially, in this process, DCs are complementing the macrophage population. In infection, recent report demonstrated that necroptosis of KCs was necessary to induce the Th2 mediated tissue repair (106) that remains to be tested to affect fibrosis resolution in the future. Equally important is the more detailed understanding of the factors involved in the switch from the pro-inflammatory to the restorative macrophage population.

Despite of the significant amount of data available in mice, we have just limited understanding about the course of events in human liver diseases. It will need future studies to analyze DC, monocyte, and macrophage populations within human liver samples not only phenotypically and functionally but also on genomic level in comparison with their murine counterparts. This can lead to better understanding of liver diseases but also for identifying novel therapeutic targets. A promising clinical perspective is to target chemokines in the early phase of the liver response to avoid inflammatory cell recruitment and further inflammation. One possibility is affecting the CCL2 axis. Currently, Cenicriviroc, an inhibitor of CCR2, is tested (Centaur study, phase 2 clinical trial, NCT:022117475) to attenuate fibrosis progression in NASH patients. Along this line, other chemokines that could affect the differentiation of monocytes to inflammatory macrophages could be a possible target in the future. Additionally, DCs and restorative macrophages could become novel objectives for inducing fibrolysis and reversing liver damage. Notably, autologous transfer of expanded mononuclear cells to chronic viral hepatitis-associated fibrotic patients showed improved outcome as indicated by reduced Child-Pugh score (122), suggesting a great potential of myeloid cell transfer-based therapeutic procedures in the future.

## Author Contributions

CE, NK contributed to the writing of the manuscript, prepared the tables. MK supervised the students and critically read the manuscript. VL-K developed the concept of the manuscript, supervised, and wrote the manuscript.

## Conflict of Interest Statement

The authors declare that the research was conducted in the absence of any commercial or financial relationships that could be construed as a potential conflict of interest.

## Abbreviations

APC, antigen presenting cell; BDL, bile duct ligation; BM, bone marrow; DCs, dendritic cells; Batf3, basic leucine zipper transcription factor, ATF-like 3; CCL_4_, carbon tetrachloride; CCR9, chemokine (C–C motif) receptor 9; CTL, cytotoxic T lymphocyte; DNGR-1, DC NK lectin group receptor-1; DT, diphtheria toxin; DTR, diphtheria toxin receptor; ECM, extracellular matrix; FA, fatty acid; Flt3L, FMS-like tyrosine kinase 3 ligand; GM-CSF, granulocyte macrophage colony-stimulating factor; HSC, hepatic stellate cells; HL-DC, high-lipid liver dendritic cell; Id2, inhibitor of DNA binding 2; IL, interleukin; KC, Kupffer cells; LL-DC, low-lipid liver dendritic cell; LSEC, liver sinusoidal endothelial cells; LPS, lipopolysaccharide; MCD, methionine choline deficient; MCP-1, monocyte chemotactic protein-1; MMP-9, matrix metalloproteinase-9; MMP13, matrix metalloproteinase-13; NASH, non-alcoholic steatohepatitis; pDC, plasmacytoid dendritic cell; PDL-1, programed cell death 1 ligand; TNFα, tumor necrosis factor-alpha; TAA, thioacetamide; TLR, toll like receptor; zbtb46, zinc finger and BTB domain containing.
